# Mental Health during the Interpregnancy Period and the Association with Pre-Pregnancy Body Mass Index and Body Composition: Data from the INTER-ACT Randomized Controlled Trial

**DOI:** 10.3390/nu15143152

**Published:** 2023-07-14

**Authors:** Hanne Van Uytsel, Lieveke Ameye, Roland Devlieger, Yves Jacquemyn, Caroline Van Holsbeke, Annick Schreurs, Annick Bogaerts

**Affiliations:** 1 REALIFE Research Group, Research Unit Woman and Child, Department of Development and Regeneration, KU Leuven, 3000 Leuven, Belgium; 2 Department of Obstetrics and Gynecology, University Hospital Leuven, 3000 Leuven, Belgium; 3 Department of Obstetrics and Gynecology, GZA Hospitals Sint-Augustinus, 2610 Antwerp, Belgium; 4 Global Health Institute (GHI), Faculty of Medicine and Health Sciences, University of Antwerp, 2610 Antwerp, Belgium; 5 Department of Obstetrics and Gynecology, University Hospital Antwerp, 2650 Antwerp, Belgium; 6 Department of Obstetrics and Gynecology, Hospital Oost-Limburg, 3600 Genk, Belgium; 7 Department of Obstetrics and Gynecology, Jessa Hospital, 3500 Hasselt, Belgium; 8 Centre for Research and Innovation in Care (CRIC), Faculty of Medicine and Health Sciences, University of Antwerp, 2610 Antwerp, Belgium; 9 Faculty of Health, University of Plymouth, Devon PL4 8AA, UK

**Keywords:** anxiety, depressive symptoms, sense of coherence, quality of life, sleep, interpregnancy, preconception

## Abstract

Mental health problems and obesity are two common complications during pregnancy and postpartum. The preconception period is considered an appropriate period for prevention. Therefore, insights into interpregnancy mental health and the impact on weight and body composition are of interest to developing effective weight management strategies. The primary aim of this study is to assess the difference in women’s mental health during the interpregnancy period and the association with pre-pregnancy body mass index (BMI) and body composition. The secondary aim is to study whether this association is affected by socio-demographic factors, interpregnancy interval and sleep. The study is a secondary analysis of the INTER-ACT e-health-supported lifestyle trial. Women were eligible if they had a subsequent pregnancy and mental health measurements at 6 weeks after childbirth and at the start of the next pregnancy (*n* = 276). We used univariate analyses to assess differences in mental health and performed regression analysis to assess their association with pre-pregnancy BMI and body composition at the start of the next pregnancy. Our results show a statistically significant increase in anxiety and depressive symptoms between 6 weeks after childbirth and the start of the next pregnancy (sSTAI-6 ≥ 40: +13%, *p* =≤ 0.001; GMDS ≥ 13: +9%, *p* = 0.01). Of the women who were not anxious at 6 weeks after childbirth (sSTAI < 40), more than one-third (39%) developed anxiety at the start of the next pregnancy (*p* =≤ 0.001). Regression analysis showed that sense of coherence (SOC-13) at the start of the next pregnancy was independently associated with women’s pre-pregnancy BMI and fat percentage. We believe that the development of preconception lifestyle interventions that focus on both weight reduction and support in understanding, managing and giving meaning to stressful events (sense of coherence) may be of added value in optimizing women’s preconception health.

## 1. Introduction

In high-income countries, it is estimated that 1 in 10 mothers experience perinatal mental health problems [1]. Nevertheless, perinatal depression is the most underdiagnosed obstetric complication [2,3,4], and, also, prenatal anxiety remains often undetected [5]. It is estimated that 70% of women hide or minimize their perinatal mental problems [6].

Negative maternal mental health is associated with women’s weight, body composition and sleep. Women with excessive GWG reported higher levels of maternal anxiety 2 days after childbirth compared to women with adequate GWG [7]. Women with pre-pregnancy overweight, whether or not in combination with excessive gestational weight gain (GWG), are at high risk for postpartum depression compared to their counterparts with a healthy pre-pregnancy weight [8,9,10]. Postpartum weight retention (PPWR) at 6 months is associated with higher levels of depression and anxiety from 6 months onwards [11]. Similarly, prospective cohort studies suggest that perinatal depression and anxiety are predictors of excessive GWG and PPWR [12,13,14]. Higher levels of depressive symptoms are also related to sleep disruption, and women with poor sleep are more vulnerable to experiencing perinatal depressive symptoms [15]. In the long term, negative mental health during the peripartum period can result in the development of chronic mental disorders, in both mother and child.

Prenatal lifestyle interventions focusing on diet and physical activity showed a positive impact on reducing GWG; however, the effect on the reduction in perinatal complications (e.g., diabetes and pregnancy-induced hypertension) is limited [16,17,18]. Previous research showed that a period between 2 and 8 months should be taken into account to build new behavior into daily routines [19], so behavioral changes take time. This may indicate that lifestyle interventions may create more impact if they start during the preconception period. Another possible reason for low lifestyle intervention effects may be the lack of attention to women’s mental health [20,21]. Mental health outcomes such as depression, anxiety, sense of coherence (SOC) and quality of life are hardly evaluated in lifestyle interventions [16,17,20,22,23]. As poor sleep is also significantly associated with gestational diabetes and pregnancy-induced hypertension, addressing women’s sleep behavior in lifestyle interventions can have a mediating effect on outcomes as well [15]. These findings suggest that lifestyle interventions focusing on weight management also need to address other related relevant health care behaviors including maternal mental health.

The interpregnancy period, which starts after childbirth and ends at the start of the next pregnancy, is an opportune time to address maternal mental health problems, as a history of depression and anxiety is a significant risk factor for postpartum depression [10,24]. Furthermore, the interpregnancy period offers an innovative window of opportunity to achieve behavioral change.

To the best of our knowledge, no studies have investigated the changes in mental health during the interpregnancy period in women with excessive GWG. Insight into the changes in interpregnancy mental health and the impact on weight and body composition can assist in the development of effective and timely weight management strategies in women at risk. The primary aim of this study is to assess the difference in mental health between 6 weeks after childbirth and the start of the next pregnancy and investigate its association with pre-pregnancy weight and body composition. The secondary aim is to study whether this association is affected by socio-demographic factors, interpregnancy interval and sleep.

## 2. Materials and Methods

The interpregnancy coaching for a healthy future (INTER-ACT) (ClinicalTrials.gov; NCT02989142) intervention is a combined e-health-supported and face-to-face coaching program, from childbirth to the end of the next pregnancy, in women with excessive gestational weight gain in the previous pregnancy. The primary aim of the study was to assess the effectiveness of the INTER-ACT intervention on pregnancy and birth-related complications (a composite outcome: gestational diabetes, pregnancy-induced hypertension, cesarean section and large-gestational-age babies) in the subsequent pregnancy. Secondary outcomes (postpartum maternal mental health, postpartum weight retention and body composition and postpartum lifestyle behaviors such as eating behavior and physical activity) have already been analyzed [25,26,27,28,29]. The current secondary analyses focus on maternal mental health, pre-pregnancy weight and body composition during the interpregnancy period. Details of the INTER-ACT randomized controlled trial (RCT) are available elsewhere [30].

### 2.1. Study Design

The INTER-ACT RCT was a multicenter RCT with a longitudinal study design. Participants received the first study visit 6 weeks after childbirth. The intervention group received the next study visit on week 12 and months 6, 12, 18, 24 and 30 after childbirth and the control group at months 6, 12, 18, 24 and 30. Once participants were pregnant, the visits on previous dates were discontinued and replaced with a pregnancy visit in the first, second and third trimesters. The intervention group received 4 e-health-supported face-to-face coaching sessions during the first 6 months after childbirth in addition to usual care (weeks 6, 8 and 12 and month 6). The control group received usual care only.

To address the current research question, we focused on the group of women who started a subsequent pregnancy and who completed mental health questionnaires at 6 weeks after childbirth and at the start of the next pregnancy.

The study was conducted according to the guidelines of the Declaration of Helsinki and was approved on 9 March 2017 by the Clinical Trial Centre/Ethical Committee UZ Leuven (protocol code B322201730956/S59889). All participants confirmed their participation by written informed consent.

### 2.2. Participants

Participants were enrolled in 6 Flemish hospitals between May 2017 and April 2019. Women were informed and recruited by trained study nurses 2 to 3 days after childbirth if they had excessive GWG according to the 2009 National Academy of Medicine (NAM) guideline [31], were aged ≥18 years and were Dutch-speaking. Women who had a twin birth, pre-pregnancy underweight (body mass index (BMI) < 18.5), had previous or planned bariatric surgery or suffered from diabetes mellitus, kidney disease, a mental disorder or stillbirth were excluded.

**Figure 1:**
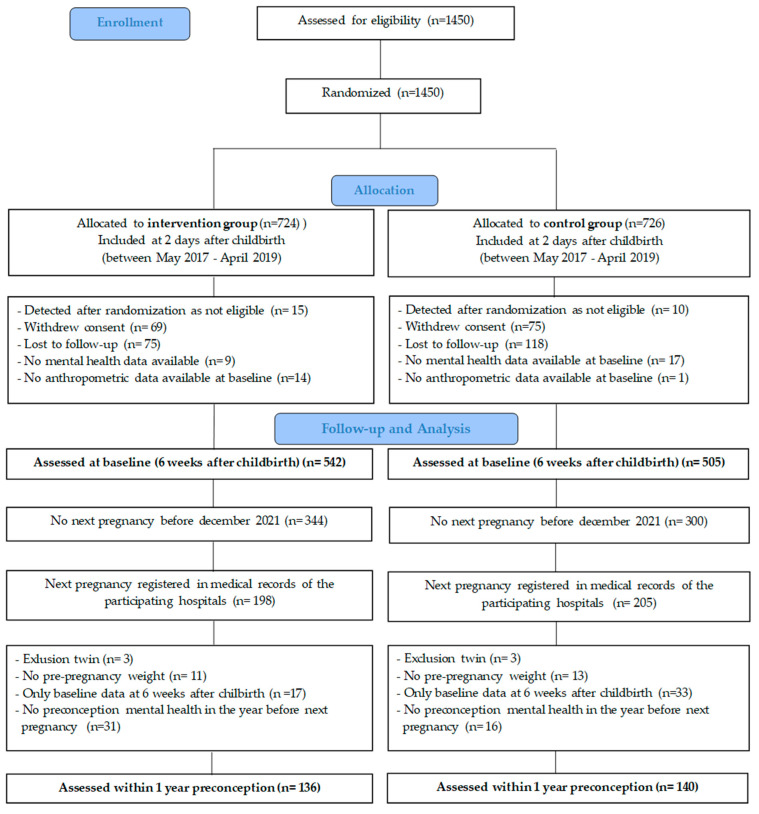
Flow chart of participant follow-up.

A total of 1450 women were enrolled and registered in the electronic case report form (eCRF) system ‘Castor’ (https://data.castoredc.com/, accessed on 20 June 2023). Randomization to the intervention or control group was performed by the biostatistician within the first week after childbirth, using an allocation ratio of 1:1 supported by block randomization (block sizes 4, 6 and 8) and stratification by hospital. Finally, 1450 women were recruited, and 1047 women were assessed at baseline (6 weeks after childbirth) (i.e., completed mental health questionnaire and body measurements). Subsequently, 403 women were registered in the medical records of the participating hospitals with a subsequent pregnancy. Of these, 6 women were excluded because of twin pregnancies, and 121 women were excluded because of missing data at the start of the next pregnancy (no mental health measurement at the start of the next pregnancy or no pre-pregnancy weight reported). A total of 276 women reported pre-pregnancy weight and completed at least 2 mental health questionnaires, 1 at 6 weeks after childbirth (start interpregnancy) and 1 at the start of the next pregnancy (end of interpregnancy) The start of the next pregnancy is defined as the timepoint nearest to the start of the next pregnancy and at least within 1 year preconception. These women constituted the study population of the current analyses. More details are shown in Figure 1.

### 2.3. Measurements

All participants were assessed at 6 weeks after childbirth and at the start of the next pregnancy by completing self-reported questionnaires. A personal link to the questionnaire was sent from the eCRF two weeks before the scheduled study visit.

Mental health was assessed by using the Edinburgh Postnatal Depression Scale (EPDS) and the Gotland Male Depression Scale (GMDS) for symptoms of depression, the Spielberger State Trait Anxiety Inventory 6-item (sSTAI-6) and the Edinburgh Depression Scale-3 Anxiety item (EDS-3A) for symptoms of anxiety, the Sense Of Coherence-13item (SOC-13) for measuring the sense of coherence and a Linear Analogue Scale (LAS) for measuring quality of life (QoL). Characteristics and cut-off scores of the mental health questionnaires are presented in Table 1.

The EPDS is suitable for assessing symptoms of depression and anxiety during the postpartum period [32]. A cut-off score of ≥10 is recommended for the detection of mild to severe postpartum depression [33]. The questionnaire included one question about self-harming. The generation of an integrated alert in the eCRF system ‘Castor’ allowed the research staff to respond immediately if this question was completed in the affirmative.

The GMDS complemented the EPDS as it is a valid instrument for assessing non-typical suicidality-related symptoms of depression. The use of the GMDS as a supplement on the GMDS can increase the detection rate of depressive symptoms [34,35]. Cut-off scores for GMDS were defined in 3 classes: <13 = no signs of depression; 13–26 indicates possible major depression; and ≥27 clearly indicates depression [36]. The sSTAI-6 [37] is a validated, reliable measure of maternal postpartum anxiety [38]. We used the shortened validated 6-item version of the sSTAI to make it less time consuming for participants. A cut-off score of 40 is commonly used to predict postpartum anxiety and mood disorder with the original 20-item questionnaire [38,39]. Therefore, the numeric sum score of the 6 items was converted to a range between 20 and 80 by using the following formula [40]: 6-item sum score/6 × 20.

Previous research indicates that three questions from the EPDS questionnaire are also sensitive to the measurement of anxiety [41]. The three items (3–4–5) are brought together under the EDS-3A. For the EDS-3A, we used a cut-off score of 5 due to the specificity of 90% and less misclassification than with a lower cut-off [41].

The SOC is measured with the 13-item short scale (SOC-13), derived from the original 29-item Orientation to Life Questionnaire [42]. For the current analysis, the format of the Institute for Data Collection and Research (centERdata) was used [43]. A SOC score <70 indicates that participants are less able to understand, influence and make sense of situations, while a high sense of coherence (≥70) indicates a better ability to cope with stressful situations in life.

QoL was assessed using the Linear Analogue Scale (LAS). Participants were asked to score QoL on a scale from 0 to 100, with 0 representing a poor quality of life and 100 an extremely good quality of life. The LAS was one comprehensive score, summarizing physical, psychological and social aspects.

Age, level of education, ethnicity, employment status, family composition, family income, a history of depression and a history of anxiety were self-reported at baseline. Questions on breastfeeding and maternal sleep were based on previous research and self-reported at each time point [44,45,46,47]. Pregnancy- and birth-related data from the first pregnancy were extracted from the medical records by research nurses at the time of recruitment.

Interpregnancy mental health was calculated based on 2 measurement points: 6 weeks after childbirth (start interpregnancy) and at the start of the next pregnancy (end of interpregnancy, defined as the timepoint nearest to the start of the next pregnancy and at least within 1 year preconception).

The difference in mental health during the interpregnancy period was calculated as the difference between mental health at 6 weeks after childbirth vs. at the start of the next pregnancy for continuous variables and as a change of group according to the cut-off score (high/low) at 6 weeks after childbirth vs. the start of the next pregnancy for categorical variables.

Interpregnancy interval was determined by calculating the difference in months between the start date of the next pregnancy (day of childbirth minus gestational age) minus the date of the previous childbirth.

### 2.4. Outcomes

Study nurses collected the self-reported pre-pregnancy weight before the previous pregnancy from the medical records 2 to 3 days after childbirth, and the self-reported pre-pregnancy weight of the next pregnancy was questioned by the INTER-ACT coach at the first INTER-ACT pregnancy visit. Body composition was electronically measured by using a Tanita MC 780 SMA bio-electric impedance (BIA) device (Tanita Corporation, Tokyo, Japan) with three frequencies (5, 50 and 250 KHz). BIA is a non-invasive, reliable and safe clinical approach, which is well accepted by patients [48]. BIA showed excellent repeatability even in pregnancy [49] and is appropriate for revealing interactions between fat mass and adverse effects in mothers. During the postpartum period, the BIA method may be of added value in the prediction of future health care problems such as obesity and diabetes type 2 [48] and is suitable to detect changes in fat mass over time [50].

Waist circumference was assessed (rounded to 0.1 cm) by using a Seca 201 tape (Seca, Hamburg, Germany) and defined as the midway between the lowest rib and the hip bone [51]. All research staff received intensive training to ensure adequate and consistent measurements. The measurement was repeated 3 times, and the mean score was reported. In case of deviations (˃0.5 cm), the measurement was repeated until agreement was obtained. 

BMI was calculated as weight (kg)/height × height (m^2^). Height was measured (rounded to 0.1cm) at the first study visit using a Seca 213 stadiometer (Seca, Hamburg, Germany), while women were standing upright with the head in the Frankfurt plane position [52].

The body measurements took place in the home of the participant, in hospital or elsewhere according to the preference of the participant.

### 2.5. Data Analyses

Statistical analyses were performed by using Statistical Package for the Social Science (SPSS) version 27.0 (IBM, Armonk, New York, NY, USA) and Statistical Analysis System (SAS) version 9.4 (Cary, New York, NY, USA).

The Kolmogorov–Smirnov test in addition to plots and histograms was used to assess the normality of distribution of continuous variables. All mental health variables showed a skewed distribution and were therefore analyzed by using non-parametric tests.

Descriptive characteristics were presented as mean and standard deviation or median and interquartile range for continuous variables and frequencies and percentages for categorical variables. To assess differences in participant characteristics between two groups, the unpaired *t*-test was used for continuous variables with a normal distribution and the Mann–Whitney U test for continuous variables with a skewed distribution. To assess differences in a continuous variable between 3 groups or more, the Kruskal–Wallis test was used. The likelihood ratio chi-square test, Fisher exact test (if expected cell count ≤10) or the linear-by-linear association chi-square was used to assess differences between categorical variables.

To assess the difference in mental health at 2 different time points in the same patients, the Mc-Nemar test was used for categorical variables and the Wilcoxon signed ranked test for continuous variables.

Participants were divided into low/high according to the pre-defined cut-off (Table 1), assessing whether or not participants changed from the cut-off group between 6 weeks after childbirth and the start of the next pregnancy (group 1: no symptoms of anxiety or depression, low SOC or low QoL at 6 weeks after childbirth or at the start of the next pregnancy; group 2: symptoms of anxiety or depression, low SOC or low QoL at 6 weeks after childbirth as well as at the start of the next pregnancy; group 3: no symptoms of anxiety or depression, low SOC or low QoL at 6 weeks after childbirth but symptoms of anxiety or depression, low SOC or low QoL at the start of the next pregnancy; group 4: symptoms of anxiety or depression, low SOC or low QoL at 6 weeks after childbirth but no symptoms of anxiety or depression, low SOC or low QoL at the start of the next pregnancy). If there was more than 1 measurement (mental health or body composition) within the year preconception, the measurement nearest to the next pregnancy was taken to conduct the analyses.

We performed regression analyses to assess whether mental health at the start and end of the interpregnancy interval were independent factors associated with BMI and body composition at the start of the next pregnancy. The outcome variables were postpartum weight retention, change in BMI at the start of each pregnancy and body composition at the start of the second pregnancy (BMI, fat percentage, waist circumference and visceral fat). We considered the mental health variables as categorical variables: high/low at the start, high/low at the end and the four possible combinations (high at start and end, high at start but low at end, etc.). We took into account as explanatory variables the following: pre-pregnancy BMI at the start of the previous pregnancy (continuous variable), level of education, exclusive breastfeeding at 6 months, interpregnancy interval (short (≤18 months) vs. normal (18–59 months)), sleep, history of depression, history of anxiety and mental health variables). Stepwise variable selection was performed to assess whether the mental health variables showed independent statistical significance to the outcome variables (BMI and body composition at the start of the next pregnancy).

## 3. Results

### 3.1. Participant Characteristics

Of the 1450 women randomized in the INTER-ACT trial, 276 women had a next pregnancy and complete data (Figure 1): mean age of 30 years (SD ± 3.6), 56% normal BMI at the start of the previous pregnancy and 42% had at least a master’s degree. Table S1 assesses the differences between these 276 patients and the patients who dropped out (Table S1).

At baseline (6 weeks after childbirth), a difference in QoL was found between the intervention and control group (median score; 80 versus 81, *p* = 0.04). No statistically significant differences were found in pre-pregnancy weight (both previous pregnancy and next pregnancy), mental health at the start of the next pregnancy (sSTAI-6, EDS-3A, EPDS, GMDS, SOC-13 and QoL) or mental health at the end of the intervention (6 months postpartum) between the intervention and control group (Table S2). Therefore, no further analyses stratified by randomization arm were performed, and we assessed the total group of 276 participants.

### 3.2. Differences in Mental Health between 6 Weeks after Childbirth and Start of Next Pregnancy

**Figure 2:**
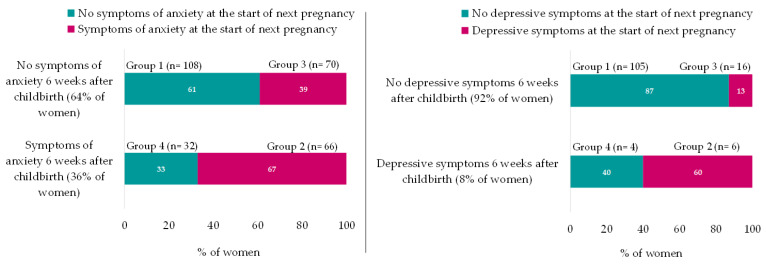
Differences in sSTAI-6 and GMDS during the interpregnancy period. sSTAI-6 N = 276; GMDS N = 131; Symptoms of anxiety = sSTAI-6 ≥ 40; depressive symptoms = GMDS ≥ 13; and GMDS was recoded from 3 to 2 categories (<13 and ≥13) because of only 1 case in category 3. group 1 = No symptoms of anxiety at 6 weeks after childbirth and no symptoms of anxiety at the start of next pregnancy; group 2 = Symptoms of anxiety at 6 weeks after childbirth and still symptoms of anxiety at the start of next pregnancy; group 3 = No symptoms of anxiety at 6 weeks after childbirth but symptoms of anxiety at the start of next pregnancy; and group 4 = Symptoms of anxiety at 6 weeks after childbirth but no symptoms of anxiety at the start of next pregnancy.

The rate of women with symptoms of anxiety (sSTAI-6 ≥ 40) increased by 13% between 6 weeks after childbirth and the start of the next pregnancy (36% vs. 49%, *p* =≤ 0.001). Of the women who were not anxious (sSTAI < 40) at 6 weeks after childbirth, more than one-third (39%) developed anxiety (sSTAI ≥ 40) by the start of the next pregnancy. Of those who were anxious at 6 weeks after childbirth (sSTAI ≥ 40), 67% still experienced anxiety at the start of the next pregnancy (sSTAI ≥ 40) (*p* =≤ 0.001) (Figure 2).

Also, the rate of women who reported depressive symptoms at the start of the next pregnancy doubled compared to 6 weeks after childbirth (GMDS ≥ 13; 17% versus 8% respectively, *p* = 0.01).

Of the women who experienced depressive symptoms (GMDS ≥ 13) at 6 weeks after childbirth (*n* = 10), 60% of women still reported depressive symptoms (GMDS ≥ 13) at the start of the next pregnancy. In the group of women without depressive symptoms at 6 weeks after childbirth, 13% of women reported depressive symptoms at the start of the next pregnancy (*p* = 0.01) (Figure 2).

No statistically significant changes were shown for differences in EDS-3A, EPDS, SOC or QoL between 6 weeks after childbirth and the end of the next pregnancy (*p* = 0.37; 1; 0.29 and 0.91, respectively).

### 3.3. Mental-Health-Related Characteristics

#### 3.3.1. Socio-Demographic Factors and Interpregnancy Interval

Women with a history of depressive feelings or a history of anxiety feelings reported more often depressive symptoms (EPDS ≥ 10, GMDS ≥ 13), anxiety (sSTAI-6 ≥ 40, EDS-3A ≥ 5) or a low SOC (SOC-13 ≤ 70) at the start of the next pregnancy. Also, women with obesity before the start of the previous pregnancy reported more common depressive symptoms (EPDS ≥ 10, GMDS ≥ 13) and anxiety (sSTAI-6 ≥ 40, EDS-3A ≥ 5) at the start of the next pregnancy compared to healthy weight or overweight women. Details are presented in Table S3.

There was no further statistically significant association between mental health at the start of the next pregnancy (sSTAI-6, EDS-3A, EPDS, GMDS, SOC, QoL or the interpregnancy differences in anxiety (STAI-6; group 1–4)) and parity, level of education, employment status, ethnicity, method of delivery and family composition (*p* = 0.35; 0.16; 0.33; 0.06; 0.97; and 0.94, respectively).

There was no association between the length of the interpregnancy interval and maternal mental health at 6 weeks after childbirth or at the start of the next pregnancy.

#### 3.3.2. Sleep

**Figure 3:**
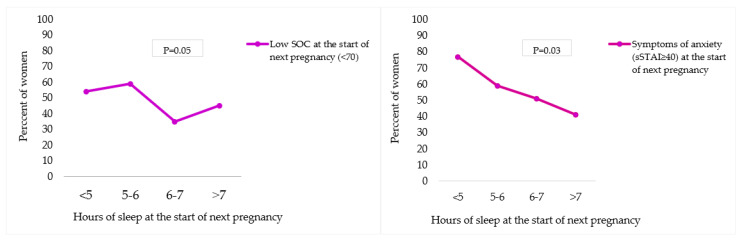
Association between sleep duration and SOC/anxiety at the start of next pregnancy. sSTAI-6 = Spielberger State-Trait Anxiety Inventory 6-item. Significance level was calculated using the Pearson Chi-Square test.

Women with less than 6 h of sleep per night at the start of the next pregnancy more often reported a low SOC (SOC < 70; 5 h or less = 54%; 5–6 h = 59%) compared to women with more than 6 h of sleep per night (SOC < 70; 6–7 h = 35% and more than 7 h = 45%) (*p* = 0.05). Also, women more often reported levels of anxiety (sSTAI-6 ≥ 40) if they obtained less than 5 h of sleep per night at the start of the next pregnancy (77%), compared to women with 5–6 h (59%), 6–7 h (51%) or more than 7 h of sleep per night (41%) (*p* = 0.03) Figure 3.

### 3.4. Association with Pre-Pregnancy BMI and Body Composition

Regression analyses showed that when taking into account pre-pregnancy BMI of the previous pregnancy, level of education and breastfeeding, only sense of coherence at the start of the next pregnancy was independently associated with women’s BMI and fat percentage at the start of the next pregnancy and BMI change between two pregnancies (Table 2).

## 4. Discussion

We showed a significant increase in anxiety (+13%, *p* =≤ 0.001) and depressive symptoms (+9%, *p* = 0.01) throughout the interpregnancy period. Remarkably, of the women who were not anxious at 6 weeks after the previous childbirth, 39% evolved to experience levels of anxiety at the start of the next pregnancy (*p* =≤ 0.001). Women with a history of depression, a history of anxiety or a pre-pregnancy BMI ≥ 30 were more vulnerable to report symptoms of anxiety and depression at the start of the next pregnancy (Table S3). Also, women with less than 6 h of sleep per night were more likely to report low SOC (*p* = 0.05) or symptoms of anxiety (*p* = 0.03) at the start of the next pregnancy, compared to women with more than 6 h of sleep per night. Furthermore, from our multivariate regression analysis, we showed that sense of coherence was independently associated with women’s BMI and fat percentage at the start of the next pregnancy (Table 2).

In contrast to the STAI-6 (symptoms of anxiety), sense of coherence is a personality trait that indicates the ability to understand, manage and give meaning to situations. It increases resistance to stress and promotes the person’s development [53]. The sSTAI-6, on the other hand, represents a feeling linked to a specific situation at a specific moment in time [37]. Our results showed a significant increase in sSTAI-6 between 6 weeks after childbirth and the start of the next pregnancy (+13%, *p* =≤0.001), suggesting that specific events related to a new pregnancy or due to postpartum events can cause changes in anxiety, while the effect on weight and fat percentage at the start of the next pregnancy appears to be significantly related to a person’s ability to cope with stressful daily events. As pre-pregnancy weight is an important risk factor for several health problems during and after pregnancy, it seems important to consider preconception interventions that focus on coping strategies that empower women to cope with stressful daily situations.

We also found that symptoms of anxiety and low SOC at the start of the next pregnancy were significantly more common in women with less than 6 h of sleep per night. However, studies investigating sleep during the preconception period and its impact on mother and child outcomes are rather scarce [54,55]. Moreover, preconception care guidelines unfortunately do not take into account interventions focusing on sleep within preconception care [56,57]. Future randomized controlled trials need to focus on the improvement of sleep quantity during the preconception period and study the impact on women’s mental health as a possible relevant and important mediator for maternal weight management. Since both anxiety and sense of coherence appear to be associated with sleep duration, preconception interventions should not only target the causes of anxiety during preconception but should also target coping strategies to enhance women’s ability to cope with stressful situations, as the latter was a significant predictor for women’s preconception BMI and fat percentage.

A remarkably high prevalence (49%) of women who experienced higher levels of anxiety (sSTAI-6 ≥ 40) at the start of the next pregnancy was shown. Most importantly, two-thirds of these women had already high levels of anxiety at 6 weeks after childbirth (i.e., during the postpartum period of the previous pregnancy), without improvement at the next pregnancy. Similarly, one-third of women who were not anxious at 6 weeks after childbirth evolved to experience levels of anxiety at the start of the next pregnancy. A possible reason for the high prevalence of levels of anxiety and depression could be the fact that we only included women with excessive GWG in a previous pregnancy, of which more than 50% were women with overweight/obesity. From recent systematic reviews, it is shown that a high maternal BMI is associated with higher levels of anxiety and depression [7,11,58]. Research showed that unfavorable mental health at the start of the next pregnancy is associated with adverse outcomes such as excessive GWG [14], maternal mental health problems after childbirth [59], low birth weight [60] and a disturbed mother–child bonding [61]. In addition, our results showed that mental health problems at the start of the next pregnancy were more common in women who had obesity, in women with less sleep and in women with a history of anxiety or depression. A standard mental health screening throughout the interpregnancy period for this population at risk could be recommended. If symptoms of depression, anxiety, low SOC or low QoL are present, timely mental health support with long-term follow-up throughout the interpregnancy period could be indicated. This may lead to benefits for mother and child and significantly less health costs in the longer term. Intervention studies on the effect of standard mental health screening during the interpregnancy/preconception period would be appropriate, as, to the best of our knowledge, these do not yet exist.

Women who dropped out of the study were more likely to be living with overweight or obesity before the previous pregnancy and reported worse mental health scores at 6 weeks after childbirth (Table S1). We assume that due to the high drop-out rate of vulnerable women, our results cannot be generalized to the total population of women in the interpregnancy period. Further research into improving the involvement of vulnerable women during the interpregnancy period could be of added value in the improvement of preconception care pathways.

A statistically significant increase in levels of depression during the interpregnancy period was shown using the GMDS questionnaire. However, this finding was not supported by the EPDS questionnaire, which is a validated and reliable scale to measure depressive symptoms in women after childbirth [32]. Further research on the validity of the GMDS for detecting levels of depression in women after childbirth is recommended.

The strength of our study was the use of data from a large longitudinal prospective randomized controlled trial. Furthermore, we analyzed four different mental health outcomes. Including SOC and QoL in addition to depression and anxiety provided new insights and added value to the knowledge of the overall concept of mental health [62]. A further strength is the use of body composition measurement in addition to weight, as visceral fat and fat and muscle mass are strong markers for women’s global metabolic health [63].

Our study also has some limitations. Firstly, our study included only women with previous excessive GWG, which is, in general, prevalent in 40–50% of women [22,64]. Excessive GWG is one of the most important risk factors for PPWR [17]. This may have an impact on the presented rate of women with PPWR during the preconception period. Secondly, the current analyses focused on women who reported a subsequent pregnancy and completed mental health measurements at baseline (6 weeks after childbirth) and at the start of the next pregnancy. Therefore, selection bias must be taken into account when interpreting and generalizing our study results to the entire population of mothers. A third limitation was that the BIA measurements could not be performed according to the standard guidelines [48]. Since our studied population consisted of postpartum women, it was not recommended that breastfeeding mothers fast for 4 h, nor was physical activity assessed in the 12 h prior to the BIA measurement, so our results could not be controlled for this. A fourth limitation was that the GMDS was added later in the study. This resulted in small groups (group 4: *n* = 4; group 2: *n* = 6) when we studied differences in GMDS during the interpregnancy period (Figure 3). Therefore, further analyses of differences in levels of depression (GMDS) on related characteristics, weight and body composition were not conducted, except for the regression analyses. A last limitation was the use of self-reported mental health questionnaires and self-reported pre-pregnancy weight. Shame and minimalization in new mothers may have led to underreporting of mental health problems during the interpregnancy period. Self-reported pre-pregnancy weight is prone to errors [65] and, in some cases, underreported, compared to objective measures by professionals [66].

## 5. Conclusions

Our data show a significant increase in anxiety and depressive symptoms between the start and the end of the interpregnancy period. Of the women who were not anxious at the start, 39% experienced anxiety at the end of interpregnancy. Sense of coherence at the start of the next pregnancy was independently associated with women’s pre-pregnancy BMI and fat percentage. Our results indicate that the interpregnancy period appears to offer an opportunity to develop innovative preventative interventions. We believe that the development of preconception lifestyle interventions that focus on both weight reduction and support in understanding, managing and giving meaning to stressful events (sense of coherence) may be of added value to optimize women’s preconception health.

## Figures and Tables

**Table 1:** Characteristics of the mental health questionnaires.

	Depression		Anxiety		SOC	QoL
	EPDS	GMDS	sSTAI-6	EDS-3A	SOC-13	QoL
**Items**	10	13	6	3	13	1
**Scale**	4-point Likert Scale	4-point Likert Scale	4-point Likert Scale	4-point Likert Scale	7-point Likert Scale	Linear Analogue Scale
**Range** **numeric score**	0–30 ᵃ	0–39 ^a^	20–80 ^a^	0–9 ^a^	13–91 ^b^	0–100 ^a^
**Cut-off score**	≥10	<13 13–26 ≥27	≥40	≥5	<70	Median ^c^

EPDS = Edinburgh Postnatal Depression Scale; GMDS = Gotland Male Depression Scale; sSTAI-6 = spielberger; State-Trait Anxiety Inventory- 6 item; EDS-3A = Edinburgh Depression Scale-3 Anxiety subscale; SOC = Sense Of Coherence; QoL = Quality of Life; ^a^: The higher the score, the higher the level of depression/anxiety/ QoL; ^b^: The higher the score, the better the sense of coherence; ^c^: Cut-off is the median calculated at each individual timepoint (6 weeks after childbirth, start next pregnancy).

**Table 2:** Association between mental health (SOC, sSTAI-6, EDS-3A, EPDS, GMDS and QoL) and pre-pregnancy BMI and body composition (regression models after stepwise variable selection).

Model 1: BMI change between 2 pregnancies *			
Explanatory variables	ß	SE	*p*-value
Intercept	2.607	0.530	<0.0001
Level of education	−0.498	0.246	0.04
Sense of coherence at that start of next pregnancy	−0.527	0.211	0.01
Model 2: Pre-pregnancy BMI next pregnancy *			
Explanatory variables	ß	SE	*p*-value
Intercept	3.628	0.938	0.0001
Pre-pregnancy BMI previous pregnancy	0.965	0.027	<0.0001
Level of education	−0.530	0.248	0.03
Sense of coherence at the start of next pregnancy	−0.577	0.214	0.008
Model 3: Visceral fat at the start of the next pregnancy *			
Explanatory variables	ß	SE	*p*-value
Intercept	−8.100	0.572	<0.0001
Pre-pregnancy BMI previous pregnancy	0.465	0.019	<0.0001
Exclusive breastfeeding at 6 months after childbirth	0.401	0.176	0.02
Model 4: Waist circumference at the start of the next pregnancy *			
Explanatory variables	ß	SE	*p*-value
Intercept	33.799	3.033	<0.0001
Pre-pregnancy BMI previous pregnancy	1.878	0.092	<0.0001
Exclusive breastfeeding at 6 months after childbirth	1.830	0.835	0.03
Interpregnancy interval (months)	−2.173	0.722	0.003
Model 5: Fat percentage at the start of the next pregnancy *			
Explanatory variables	ß	SE	*p*-value
Intercept	2.039	2.254	0.367
Pre-pregnancy BMI previous pregnancy	1.130	0.066	<0.0001
Exclusive breastfeeding at 6 months after childbirth	1.952	0.609	0.002
Sense of coherence at the start of next pregnancy	−1.200	0.528	0.02

Pre-pregnancy BMI at the start of previous pregnancy (continuous variable), level of education (1 = secondary school, 2 = bachelor/master), exclusive breastfeeding at 6 months (1 = yes, 2 = no), interpregnancy interval (1 = short vs. 2 = normal) and Sense of coherence at the start of next pregnancy (continuous variable). * = Outcome variable.

## Data Availability

The study protocol is publicly available. Data requests may be submitted to the Principal Investigator accompanied by a proposal with a planned objective for use of data.

## Supplementary Materials

The following supporting information can be downloaded at: https://www.mdpi.com/article/10.3390/nu15143152/s1, Table S1: Participant characteristics; Table S2: Participant characteristics and the difference between intervention and control group; Table S3: Prevalence of symptoms of anxiety and depression, low SOC and low QoL at the end of interpregnancy in relation to participant characteristics.
