# Supplementary material for: Mental Health during the Interpregnancy Period and the Association with Pre-Pregnancy Body Mass Index and Body Composition: Data from the INTER-ACT Randomized Controlled Trial
Source: Nutrients. 2023 Jul 14;15(14):3152. doi: 10.3390/nu15143152 (PMC10384439; doi:10.3390/nu15143152)
Supplement: Supplementary file 1 [file nutrients-15-03152-s001.zip › Table S1.pdf]

Table S1. Participant characteristics

		Overall	At least 2 measurements during the interpregnancy period		P- value
			Yes	No	
		(n=1047)	(n= 276)	(n= 771)	
**Age at previous birth, mean ±SD**		31 ±4	30 ±3,6	32 ±4	**<0.001**
**Parity at baseline, n (%)**	Primiparous	572 (55)	224 (81)	348 (45)	**<0.001**
	Multiparous	475 (45)	52 (19)	423 (55)	
**Education at baseline, n (%)**	Secondary degree	300 (29)	66 (24)	234 (30)	**<0.001**
	Bachelor degree	407 (39)	93 (34)	314 (41)	
	Master degree and above	340 (32)	117 (42)	223 (29)	
**Ethnicity, n (%)**	White European	963 (92)	266 (96)	697 (90)	**0.001**
	Other ethnicity	84 (8)	10 (4)	74 (10)	
**Method of conception previous childbirth, n (%)**	Spontaneous	920 (90)	241 (91)	679 (90)	0.81
	ART	100 (10)	25 (9)	75 (10)	
	Missing	27	10	17	
**Method of delivery previous childbirth, n (%)**	Spontaneous	702 (67)	184 (67)	518 (67)	**0.04**
	Vacuum- extraction/ forceps	99 (10)	37 (13	62 (8)	
	Primary section (planned)	119 (11)	19 (7)	100 (13)	
	Secondary section (urgent)	127 (12)	36 (13)	91 (12)	
**Exclusive breastfeeding at 6 weeks postpartum, n (%)**	Yes	561 (54)	178 (64)	383 (50)	**<0.001**
	No	486 (46)	98 (36)	388 (50)	
**Family composition baseline, n (%)**	Biological parents				
	Single parent family	75 (7)	14 (5)	61 (8)	**<0.001**
	Two parent family	893 (85)	253 (92)	640 (83)	
	Blended family	79 (8)	9 (3)	70 (9)	
**Family income (monthly) at baseline, n (%)**	Less than 2000 euro	69 (7)	13 (5)	56 (8)	**0.03**
	2000-3000 euro	179 (18)	36 (14)	143 (20)	
	3000-4000 euro	454 ((46)	124 (47)	330 (45)	
	4000 or above	284 (29)	88 (34)	196 (27)	
	Missing	61	15	46	
**History of depressive feelings before previous childbirth, n (%)**	Yes	153 (15)	30 (11)	123 (17)	**0.04**
	No	850 (85)	236 (89)	614 (83)	
	Missing	44	10	34	
**History of anxiety feelings before previous childbirth, n (%)**	Yes	112 (11)	27 (10)	85 (12)	0.57

	No	888 (89)	238 (90)	650 (88)	
	Missing	47	11	36	
**Pre- pregnancy BMI previous pregnancy, n (%)**	NW *	506 (48)	154 (56)	352 (46)	**0.003**
	OW	375 (36)	89 (32)	286 (37)	
	OB	166 (16)	33 (12)	133 (17)	
**Interpregnancy Interval, n (%)**	Short (<18 months)	.	145 (53)	.	
	Normal (18 to 60 months)	.	131 (47)	.	
**GWG in kg previous pregnancy, mean (IQR)**	Among NW	19 (4)	18 (3)	19 (4)	0.33
	Among OW	16 (6)	16 (6)	16 (6)	0.76
	Among OB	14 (4)	15 (5)	13 (6)	**0.02**
**Depression score at baseline, mean (IQR)**	EPDS (0-30)	6 (6)	6 (6)	7 (6)	**<0.001**
	GMDS (0-39)	5 (6)	4 (5)	6 (6)	**0.007**
**Anxiety score at baseline, mean (IQR)**	sSTAI (20-80)	37 (13)	37 (13)	40 (13)	**<0.001**
	EDS-3A (0-9)	4 (3)	3 (4)	4 (3)	**0.02**
**QoL score at baseline, mean (IQR)**	QoL (0-100)	80 (15)	80 (12)	77 (14)	**<0.001**
**SOC score at baseline, mean (IQR)**	SOC (13-91)	70 (16)	72 (14)	69 (16)	**0.004**
**Interpregnancy Interval (months), mean (IQR)**		.	17 (11)	.	

ART = assisted reproductive treatment; BMI= body mass index; NW= normal weight; OW= overweight; OB= obesity; sSTAI-6= spielberger State-Trait Anxiety Inventory- 6 item; EDS-3A = Edinburgh Depression Scale- 3 Anxiety subscale; EPDS= Edinburgh Postnatal Depression Scale; GMDS = Gotland Male Depression Scale; SOC-13 = Sense Of Coherence 13 item; GWG= gestational weight gain; QoL= Quality of Life; SOC= Sense Of Coherence. Significance level was calculated using the Likelihood ratio chi-square test or Lineair-by-Lineair Association for categorical variables and the Mann- Whitney U test for continue variables. Baseline = 6 weeks after childbirth - Inter-Conception Interval = time between childbirth and start next pregnancy.

* Two participants (0.7%) developed underweight before the start of the next pregnancy (BMI of 16.3 and 17.9 kg/m2 respectively), however being underweight before the next pregnancy was not an exclusion criteria. These women (n=2) were included and analyzed in the group of women with a healthy pre-pregnancy BMI before the next pregnancy (N=136; 49.3% of the total study population), because of the small sample size.
