# Supplementary material for: Mental Health during the Interpregnancy Period and the Association with Pre-Pregnancy Body Mass Index and Body Composition: Data from the INTER-ACT Randomized Controlled Trial
Source: Nutrients. 2023 Jul 14;15(14):3152. doi: 10.3390/nu15143152 (PMC10384439; doi:10.3390/nu15143152)
Supplement: Supplementary file 1 [file nutrients-15-03152-s001.zip › Table S2.pdf]

Table S2. Participant characteristics and the difference between intervention- and control group

		Overall<br><br>(n= 276)	Intervention group<br><br>(n= 136)	Control group<br><br>(n= 140)	P-value
**Age at previous birth, mean ±SD**		30 ±3.6	29.4 ±3.4	30.2 ±3.4	0.51
**Parity at baseline, n (%)**	Primiparous	224 (81)	110 (81)	114 (81)	1
	Multiparous	52 (19)	26 (19)	26 (19)	
**Education at baseline, n (%)**	Secondary degree	66 (24)	31 (23)	35 (25)	0.86
	Bachelor degree	93 (34)	50 (37)	43 (31)	
	Master degree and above	117 (42)	55 (40)	62 (44)	
**Ethnicity, n (%)**	White European	266 (96)	131 (96)	135 (96)	1
	Other ethnicity	10 (4)	5 (4)	5 (4)	
**Method of conception previous childbirth, n (%)**	Spontaneous	241 (91)	112 (86)	129 (96)	**0.006**
	ART	25 (9)	19 (14)	6 (4)	
	Missing	*10*	*5*	*5*	
**Method of delivery previous childbirth, n (%)**	Spontaneous	184 (67)	86 (63)	98 (70)	0.49
	Vacuum- extraction/ forceps	37 (13)	23 (17)	14 (10)	
	Primary section (planned)	19 (7)	9 (7)	10 (7)	
	Secondary section (urgent)	36 (13)	18 (13)	18 (13)	
**Exclusive breastfeeding at 6 weeks postpartum*, n (%)**	Yes	178 (64)	80 (59)	98 (70)	**0.052**
	No	98 (36)	56 (41)	42 (30)	
**Family composition baseline, n (%)**	Biological parents				
	Single parent family	14 (5)	8 (6)	6 (4)	0.80
	Two parent family	253 (92)	124 (91)	129 (92)	
	Blended family	9 (3)	4 (3)	5 (4)	
**Family income (monthly) at baseline, n (%)**	Less than 2000 euro	13 (5)	5 (4)	8 (6)	0.53
	2000-3000 euro	36 (14)	14 (11)	22 (16)	
	3000-4000 euro	124 (47)	61 (48)	63 (47)	
	4000 or above	88 (34)	46 (37)	42 (31)	
	Missing	*15*	*10*	*5*	
**History of depressive feelings before previous childbirth, n (%)**	Yes	30 (11)	10 (8)	20 (15)	0.08
	No	236 (89)	121 (92)	115 (85)	
	Missing	*10*	*5*	*5*	
**History of anxiety feelings before previous childbirth, n (%)**	Yes	27 (10)	15 (11)	12 (9)	0.55
	No	238 (90)	116 (89)	122 (91)	
	Missing	*11*	*5*	*6*	
**Pre- pregnancy BMI previous pregnancy, n (%)**	NW	154 (56)	76 (56)	78 (56)	1
	OW	89 (32)	44 (32)	45 (32)	
	OB	33 (12)	16 (12)	17 (12)	

**Gestational weight gain in kg previous pregnancy, median (IQR)**	Among NW	18 (3)	18 (3)	19 (4)	0.11
	Among OW	16 (6)	16 (5)	16 (6)	0.41
	Among OB	15 (5)	15 (7)	14.5 (4)	0.40
**Depression score at baseline, median (IQR)**	EPDS (0-30)	6 (6)	6 (7)	5 (6)	0.49
	GMDS (0-39)	4 (5)	5 (5)	4 (6)	0.74
**Anxiety score at baseline, median (IQR)**	sSTAI (20-80)	37 (13)	35 (10)	37 (13)	0.20
	EDS-3A (0-9)	3 (4)	3 (3)	4 (3)	0.80
**Quality of life score at baseline, median (IQR)**	(0-100)	80 (12)	80 (14)	81 (11)	**0.04**
**Sense of Cohrence score at baseline, median (IQR)**	SOC (13-91)	72 (14)	71 (17)	71 (16)	0.41
**Depression score at 6 months after childbirth, median (IQR)**	EPDS (0-30)	5 (5)	5 (6)	5 (5)	0.63
	GMDS (0-39)	5 (6)	5 (6)	4 (6)	0.89
**Anxiety score at 6 months after childbirth, median (IQR)**	sSTAI (20-80)	37 (16)	40 (17)	37 (13)	0.17
	EDS-3A (0-9)	3 (2)	3 (4)	3 (2)	0.70
**Quality of life score at 6 months after childbirth, median (IQR)**	(0-100)	80 (16)	80 (14)	80 (15)	0.07
**Sense of Coherence at 6 months after childbirth, median (IQR)**	SOC (13-91)	73 (17)	74 (16)	71 (15)	0.57
**Interpregnancy Interval (months), median (IQR)**		17 (11)	17 (12)	18 (11)	0.09

pp= postpartum; ART = assisted reproductive treatment; BMI= body mass index; NW= normal weight; OW= overweight; OB= obesity; sSTAI-6= spielberger State-Trait Anxiety Inventory- 6 item; EDS-3A = Edinburgh Depression Scale- 3 Anxiety subscale; EPDS= Edinburgh Postnatal Depression Scale; GMDS = Gotland Male Depression Scale; SOC = Sense Of Coherence

Significance level was calculated using the Likelihood ratio chi- square test or Lineair-by-Lineair Association for categorical variables and the Mann- Whitney U test for continue variables.

Baseline = 6 weeks postpartum Interpregnancy Interval = time between childbirth and start next pregnancy.
