# Supplementary material for: Mental Health during the Interpregnancy Period and the Association with Pre-Pregnancy Body Mass Index and Body Composition: Data from the INTER-ACT Randomized Controlled Trial
Source: Nutrients. 2023 Jul 14;15(14):3152. doi: 10.3390/nu15143152 (PMC10384439; doi:10.3390/nu15143152)
Supplement: Supplementary file 1 [file nutrients-15-03152-s001.zip › Table S3.pdf]

Table S3. Prevalence of symptoms of anxiety and depression, low sense of coherence and low quality of life in the year before next pregnancy in relation to participant characteristics

Participant characteristics at the start of previous pregnancy			*Anxiety end of interpregnancy*						*Depression end of interpregnancy*						*SOC end of interpregnancy*			*OoL end of interpregnancy*		
			**STAI-6 ≥ 40** n= 136 (49%)			**EDS-3A ≥ 5** n= 72 (26%)			**EPDS ≥ 10** n= 67 (24%)			**GMDS* ≥ 13** n= 36 (13%)			**SOC-13 < 70** n= 120 (44%)			**QoL < 80** n= 110 (40%)		
		n total	n	%	P- value	n	%	P- value	n	%	P- value	n	%	P- value	n	%	P- value	n	%	P- value
**Parity**	Primiparous	224	111	50	0.85	61	27	0.39	56	25	0.60	27	14	0.35	98	44	0.88	87	39	0.43
	Multiparous	52	25	48		11	21		11	21		9	20		22	42		23	45	
**Education**	Secondary level	66	35	53	0.70	24	36	0.07	20	30	0.36	9	16	0.92	34	52	0.18	28	42	0.64
	Bachelor's level	93	43	46		24	26		19	20		11	13		42	45		39	43	
	Master's level or higher	117	58	50		24	21		28	24		16	15		44	38		43	37	
**Employment status**	Employed	262	128	49	0.59	67	26	0.37	62	24	0.34	32	14	0.08	112	43	0.41	103	40	0.58
	Unemployed	14	8	57		5	36		5	36		4	33		8	57		7	50	
**Method of conception**	Spontaneous	241	122	51	0.09	64	27	1	60	25	1	34	16	0.54	110	46	0.14	100	42	0.21
	ART	25	8	32		6	24		6	24		2	9		7	28		7	28	
	Missing	10	6			2			1						3			3		
**Method of delivery**	Spontaneous	184	91	50	0.96	46	25	0.23	41	22	0.34	19	12	0.23	78	42	0.83	74	41	0.58
	Vacuum- extraction/forceps	37	17	46		12	32		11	30		7	23		17	46		16	43	
	Primairy section (planned)	19	10	53		2	11		3	16		4	22		10	53		9	47	
	Secondairy section (urgent)	36	18	50		12	33		12	33		6	18		15	42		11	31	
**Family compositon**	Single parant family	14	8	57	0.80	4	29	0.93	3	21	0.86	1	8	0.75	6	43	0.90	6	43	1
	Two biological parents	253	124	49		66	26		63	25		35	15		111	44		100	40	
	New formed family	9	4	44		2	22		1	11		0	0		3	33		4	44	
**Family income (monthly)**	0-2000	13	6	46	0.26	5	39	0.19	4	31	0.67	5	29	0.14	7	54	0.90	7	54	0.31
	2000- 3000	36	22	61		10	28		10	28		3	10		15	42		17	50	
	3000-4000	124	53	43		36	29		30	24		16	14		60	48		45	36	
	4000 and above	88	44	50		16	18		17	19		10	13		28	32		32	36	
	Missing	15	11			5			6			2			10			9		
**History of depressive feelings**	Yes	30	22	73	**0.006**	15	50	**0.003**	14	47	**0.005**	8	31	**0.03**	24	80	**<0.001**	15	50	0.32
	No	236	109	46		52	22		49	21		27	13		92	39		91	39	
	Missing	10	5			5			6			1			4			4		
**History of anxiety feelings**	Yes	27	21	78	**0.002**	14	52	**0.002**	14	52	**0.001**	7	30	0.06	22	82	**<0.001**	14	54	0.15
	No	238	109	46		53	22		48	20		28	13		92	39		92	39	
	Missing	11	6			5			5			1			6			4		
**Pre pregnancy BMI**	Among NW	153	78	51	**0.03**	37	24	**0.003**	34	22	**0.001**	19	14	**0.003**	60	39	0.08	55	36	0.15
	Among OW	90	36	40		18	20		16	18		7	9		40	44		37	41	
	Among OB	33	22	67		17	52		17	52		10	37		20	61		18	55	

pp= postpartum; QoL= Quality of life; ART = assisted reproductive treatment; BMI= body mass index; NW= normal weight; OW= overweight; OB= obesity; EPDS= Edinburgh Postnatal Depression Scale; GMDS = Gotland Male Depression Scale; sSTAI-6= spielberger State-Trait Anxiety Inventory 6-item; EDS-3A = Edinburgh Depression Scale- 3 Anxiety subscale; SOC = Sense Of Coherence* GMDS was transformed from 3 to 2 categories (<13 and ≥13) because of only 1 case in category 3.Row % are represented
